# Machine learning classification of multiple sclerosis patients based on raw data from an instrumented walkway

**DOI:** 10.1186/s12938-022-00992-x

**Published:** 2022-03-30

**Authors:** Wenting Hu, Owen Combden, Xianta Jiang, Syamala Buragadda, Caitlin J. Newell, Maria C. Williams, Amber L. Critch, Michelle Ploughman

**Affiliations:** 1 Department of Computer Science, Memorial University of Newfoundland, Newfoundland, Canada; 2 Faculty of Medicine, Memorial University of Newfoundland, Newfoundland, Canada

**Keywords:** Multiple sclerosis, Gait analysis, Machine learning, Walkway, Artificial intelligence, Rehabilitation

## Abstract

**Background:**

Using embedded sensors, instrumented walkways provide clinicians with important information regarding gait disturbances. However, because raw data are summarized into standard gait variables, there may be some salient features and patterns that are ignored. Multiple sclerosis (MS) is an inflammatory neurodegenerative disease which predominantly impacts young to middle-aged adults. People with MS may experience varying degrees of gait impairments, making it a reasonable model to test contemporary machine leaning algorithms. In this study, we employ machine learning techniques applied to raw walkway data to discern MS patients from healthy controls. We achieve this goal by constructing a range of new features which supplement standard parameters to improve machine learning model performance.

**Results:**

Eleven variables from the standard gait feature set achieved the highest accuracy of 81%, precision of 95%, recall of 81%, and F1-score of 87%, using support vector machine (SVM). The inclusion of the novel features (toe direction, hull area, base of support area, foot length, foot width and foot area) increased classification accuracy by 7%, recall by 9%, and F1-score by 6%.

**Conclusions:**

The use of an instrumented walkway can generate rich data that is generally unseen by clinicians and researchers. Machine learning applied to standard gait variables can discern MS patients from healthy controls with excellent accuracy. Noteworthy, classifications are made stronger by including novel gait features (toe direction, hull area, base of support area, foot length and foot area).

## Background

Multiple sclerosis (MS) is a common inflammatory neurodegenerative disease [1] with a prevalence of 1:400 (90,000) in Canada [2]. MS symptoms, which include slower information processing, walking impairment and feelings of mental fatigue, profoundly impact a patient’s quality of life [3].

MS-related gait disorders, including spasticity, leg weakness, foot drop and ataxia, disrupt everyday tasks [4–6] and present differently from person-to-person likely because of unique central nervous system lesions and neural reorganization [1, 7]. Most studies examining gait changes in MS focus on reductionist methods, which report output variables such as walking velocity or distance walked.

Newer technologies and analysis techniques provide expanded opportunities to map the unique gait patterns within and between individuals. Such innovations help detect changes early, which may direct rehabilitation interventions to improve walking [8, 9]. For example, using image-processing techniques [10] and wearable sensors, users can create movement-related features such as standing and sitting accelerations, rotation velocity of turning and inclination degrees of the trunk in a three-dimensional coordinate system [11, 12] to detect dynamic balance and the risk of falling [13]. In most cases these methods require specialized equipment not readily available to clinicians such as inertial measurement units and electromyograms.

A standard gait analysis system employed in clinical settings involves the use of an instrumented walkway containing a dense matrix of embedded sensors to capture temporal, spatial and force-related gait data from footsteps. Depending on the subject and the length of the mat, one pass across the walkway captures 4 to 10 footsteps and can generate thousands of individual raw sensor data points. Walkway systems often use secondary software packages to transform the raw sensor data into a standard set of output variables (speed, step length, etc.) which may be useful for clinicians [14, 15]. However, by interrogating the raw data directly, subtle changes to gait patterns could reveal signs of disease progression or improvement [16]. Data-driven techniques such as machine learning classification make it possible to analyze specific gait features and their relationships with one another. For instance, Chen et al. in 2020 employed machine learning to gait variables extracted from walking and jumping tests to classify patients with mild cognitive impairment [17]. Furthermore, data gathered from vertical ground reaction force sensors provided algorithms that detected early signs of Parkinson’s disease [18]. In the field of MS, there is a study using machine learning techniques to detect which gait parameters were most sensitive to subtle changes in gait [19]. However, this study and those described above, used the predetermined, and rather limited, gait variables available in conventional proprietary software, meaning clinicians have to interpret what they need from the data.

Creating novel gait variables from raw walkway data may further increase detection accuracy, thereby specifically pinpointing the gait characteristics requiring clinical attention. This may result in more tailored, individualized, and effective rehabilitation strategies for gait training.

The purpose of this study was to employ machine learning technology, in combination with raw data obtained from an electronic walkway (Protokinetics Havertown PA), to classify subjects as an MS patient or a healthy control. We achieved this in two series of analysis using a standard set and an expanded set of features, respectively; the expanded feature set included several new or underutilized parameters derived from the raw data, including toe direction, hull area, base of support area, foot length and foot area.

We hypothesized that machine learning models can effectively distinguish MS patients from the healthy control group using only standard features, and those novel features would further improve the detection accuracy. To the best of our knowledge, this study is the first attempt to distinguish MS patients from healthy controls using machine learning of raw walkway sensor data. Such methodologies could have important implications for detecting subtle gait changes indicative of worsening or improvement of neurological impairment automatically and accurately.

## Results

Our study compares the classification metrics of two distinct feature sets when separating MS patients from healthy controls using only gait-related spatial and temporal data. Gait parameters for each feature set were calculated from the raw data provided from an instrumented walkway in a clinical setting.

The first set has been defined as the standard set and contains a collection of gait-related parameters similar to those involved in regular gait studies. This set was initialized with 11 standard parameters, which were optimized into a final set of 10 parameters for machine learning testing and training (see Table 1).

**Table 1:** Initial and final features for each feature set after feature selection

Initial standard features	Final standard features
Standard set	
Step time	
Step velocity	Step velocity
Single support time	Simple support time
Double support time	Double support time
Stance time	Stance time
Toe angle (signed)	Toe angle (signed)
Step length	Step length
Step width	Step width
Stride length	Stride length
Stride width	Stride width
Foot type	Foot type
Augmented set	
Step time	
Step velocity	Step velocity
Single support time	Single support time
Double support time	Double support time
Stance time	
Toe angle (unsigned)	Toe angle (unsigned)
Step length	Step length
Step width	Step width
Stride length	Stride length
Stride width	Stride width
Foot type	Foot type
Toe direction (in/out)	Toe direction (in/out)
Hull area	Hull area
Base of support area (BOS Area)	Base of support area (BOS area)
Line of progression deviation angle (LOP deviation angle)	
Foot length	Foot length
Foot width	Foot width
Foot area	Foot area

The second feature set, defined as the augmented set, contains the same initialization as the standard set, plus additional new parameters that were derived from the raw walkway data (see Method section for details). The classification value of these additional parameters has not been well documented in the literature, and it is likely that some are novel to the field. We began with an initial set of 18 parameters in the augmented set, which was optimized to a final set of 15 features for machine learning. Table 1 outlines the initial and optimal features selected for machine learning in each set.

**Fig. 1:**
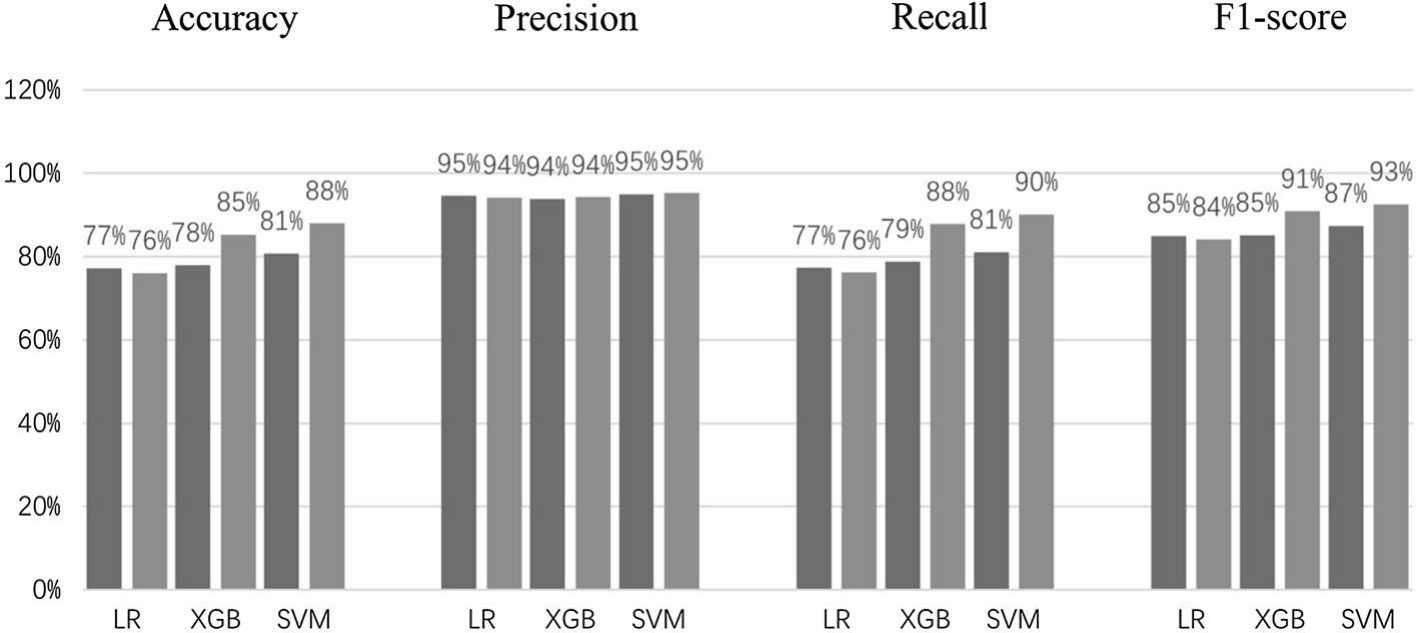
Accuracy, precision, recall and F1 score for each model. The black bars represent the standard set, while the grey bars represent the results for the augmented set

Three classification algorithms, Logistic Regression (LR), XGBoost (XGB), and Support Vector Machine (SVM), were evaluated on both feature sets. For each feature set, the accuracy, precision, recall, and F1 scores were calculated to analyze the predictive ability of each machine learning model. Figure 1 shows the classification metrics of the standard set (black) and the augmented set (grey) for the three classification algorithms, respectively.

The results outlined above show that by just using the standard set, we achieved accuracy of 81% (SVM), precision of 95% (SVM and LR), recall of 81% (SVM) and F1-score of 87% (SVM). The results also indicate a varying level of ability among the three machine learning models that were tested, with SVM providing the highest overall scores.

Worth noting are the improvements measured across all metrics when using the augmented set. This inclusion of novel features increased accuracy by 7%, recall by 9%, and F1 score of 6% from both XGB and SVM models. Notice that precision has not been improved due to the imbalanced data in the testing data set (see Table 9 for the definition of precision), where the number of false positives was relatively small compared to that of true positives.

**Fig. 2:**
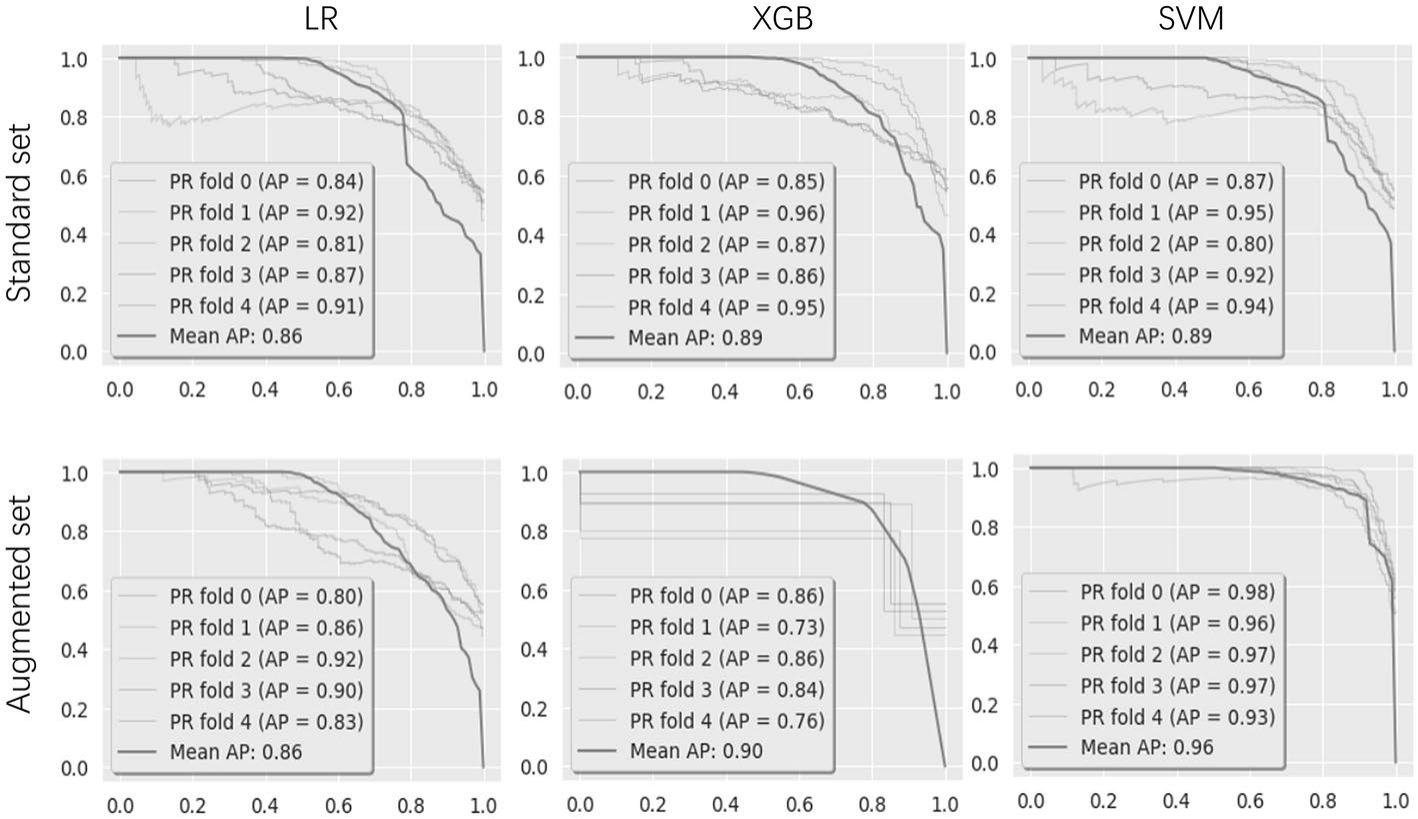
PRC curves for LR, XGB and SVM. AP refers to the area underneath the precision-recall (AUPRC)

**Fig. 3:**
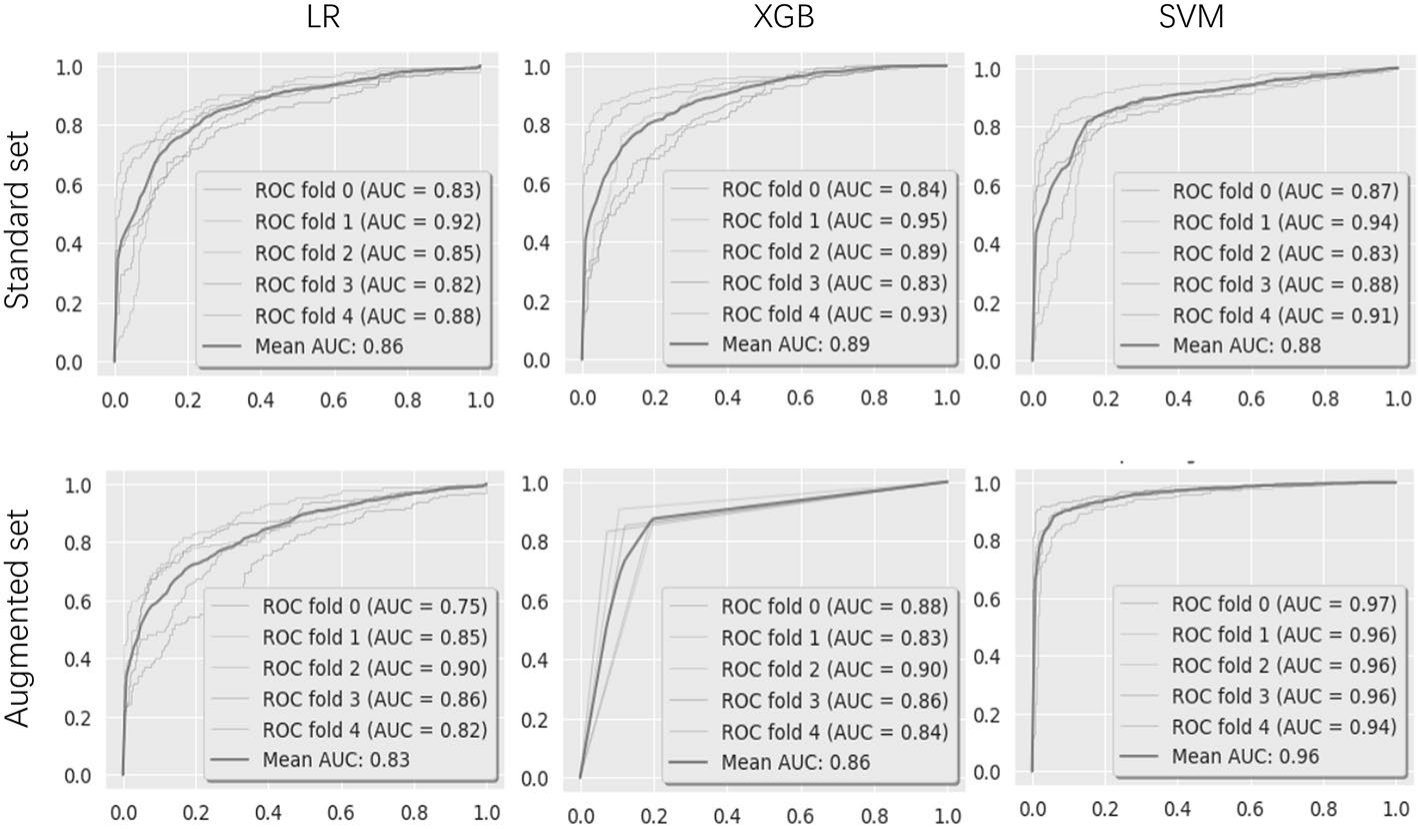
ROC curves for LR, XGB and SVM. AUC refers to the area underneath the precision-recall (AUPRC)

In addition to the scoring metrics, the area underneath the precision-recall (AUPRC) and area underneath the receiver operating characteristic (AUROC) curves were also used for determining the overall effectiveness of a classifier. Figures 2 and 3 summarize the results from these three models.

When studying the standard feature set, we achieved our best baseline of AUROC at 0.88 (XGB), and baseline of AUPRC at 0.89 (SVM). Low variance was measured between all classifiers on these scoring metrics, resulting in similar scores for all models.

The AUPRC and AUROC scoring metrics were compared for the augmented feature set as well. When using the augmented set, AUROC of LR and XGB was not improved, however, the AUROC increased when using SVM and AUPRC of all models were improved.

## Discussion

Our hypothesis was supported by the results that machine learning classifiers using raw walkway data can distinguish between persons having MS-related gait dysfunction and healthy controls. Using only the gait features extracted from the raw walkway data, the machine learning classifiers were capable of separating MS patient and control groups with an accuracy of 81%. When novel features, foot length, foot area, hull area, and BOS area were added to the dataset, the classifiers gained roughly a 7% increase in accuracy. These results demonstrate that machine learning models trained on new features from raw walkway data can more effectively separate patient and control targets and could potentially be served as an alternative method for identifying gait abnormalities in MS.

The results obtained from these experiments are notable for several reasons. Firstly, classification with high accuracy was possible using only data gathered from an instrumented walkway system [14, 15]. At present, clinicians, and patients use a wide variety of walking tests (Timed 25 Foot Walk Test, Six-Minute Walk Test, Dynamic Gait Index, 12-Item Walking scale, and others) to identify gait problems [20–22]. The machine learning process described in this paper may be useful to automatically distinguish gait problems. Future work is needed to examine performance of the classifier in longitudinal studies of gait. It is also important to determine whether the tool could be used to detect very subtle changes not easily observed by assessors.

Secondly, there is a wealth of information residing in the raw gait data that clinicians may not be taking full advantage of. Previous studies focused on the analysis of the predetermined features provided by the conventional software [19]. In contrast, the present study has shown that it is possible to design and develop new measurements of gait from raw walkway data (toe direction, hull area, BOS area, foot length and foot area). As for BOS area, this gait variable has been previously used to distinguish MS patients from healthy controls [23], however, the current project is the first to use BOS area as a feature for machine learning classification. In addition, these new measurements can provide a significant improvement in classification accuracy. Furthermore, these novel and hidden gait features may have utility as indicators of gait-related impairment that may be useful to clinicians for treatment, or to researchers who study ways to detect or delay disease progression.

Thirdly, classification based solely on gait analysis may not be restricted to impairment in MS. Gait impairment is an unfortunate side effect of many neurological diseases such as Parkinson’s disease and stroke [24–26]. This machine learning structure may be applicable in other fields of study as a relatively fast and reliable method of identifying a range of gait-related impairments. However, this study did not examine the model’s ability to distinguish patients with MS from patients with other neurological disorders such as mild cognitive impairment. Future studies could test whether the model could discern between patient groups.

The results gathered in this stage of the study are promising for the identification of subjects with gait-related dysfunction. Several improvements have been identified for future study which may further increase the usefulness of the results for gait researchers and clinicians.

The first of these involves the pre-screening of patients based on the Multiple Sclerosis Impact Scale (MSIS-29) intake survey [27, 28]. This study included only those patients who reported moderate-to-high scores (> 3 indicating moderate to severe walking problems) on the MSIS-29. Future studies could include patients who report lower scores (1 and 2) on the MSIS-29 to possibly classify patients that show milder forms of gait dysfunction.

The second improvement would involve layering kinematic data (i.e., joint angles) on top of the temporal and spatial data available from the walkway systems. This would enrich the dataset and would likely prove useful in boosting classification accuracy even further. For instance, machine learning could be useful to map changes in specific types of gait impairment such as hemiplegia or ataxia, over time.

Finally, the machine learning models would be better served with a larger dataset. Previous larger studies have proven that machine learning technology combined with gait measurements could effectively distinguish patients at cognitive impairment levels [17]. Coordinating efforts between multiple laboratories and research hospitals could result in a dataset of thousands of patients, allowing the machine learning models to train on a much richer set of underlying data and provide stronger conclusions.

## Conclusions

This paper demonstrates how machine learning can be used to classify healthy controls from persons with neurological gait impairment due to MS using only raw data collected from an instrumented walkway system. Advances in computerized machine learning and classification can easily handle the complicated underlying sensor data and make it possible for researchers to detect gait issues automatically and rapidly.

This paper has chosen to study gait by an examination of the raw underlying data. This allowed for the reconstruction of the standard gait parameters, but also for the development of new features, such as BOS area, LOP deviation angle, hull area and toe direction, for gait study. These parameters were then given to machine learning classifiers to determine the separability of MS patients and healthy controls based on gait.

The machine learning system discussed in this paper has achieved a base classification accuracy of 81% using only standard spatial and temporal gait parameters derived from the raw data. When these standard parameters were augmented with other custom parameters and normalized subject characteristics, the classification accuracy of SVM was improved to 88%. This result demonstrates that analyzing the raw gait data is a worthwhile exercise in increasing the classification accuracy of patients/healthy controls.

## Methods

### Participants and experimental protocol

Data were collected as part of the Health Innovation Team in MS (HITMS) project, a longitudinal study of the health of people with MS in Newfoundland & Labrador, Canada [29, 30]. The study was approved by the institutional health research ethics board (HREB # 2015.103). We extracted all walkway data from participants who attended between 2016 and 2019 (*n* = 126). Each patient had at least one visit and was able to walk with or without a walking assistive device [31]. Controls were required to have no walking impairments.

We then gathered demographic data for all participants (age, height, and weight). People with MS had a confirmed diagnosis by an MS neurologist who scored disease severity using the Expanded Disease Severity Scale (EDSS) [32]. The EDSS ranges from 0 to 10; 0 having no symptoms, 6 using a gait aid and 10 means death due to MS. The patients had EDSS scores from 0 (no observable gait dysfunction) to EDSS 6.5 (requires bilateral walking aids, can walk at least 20 m). The average EDSS score of all patients was 2.11 ± 1.89. At the visit, all patients completed the MSIS-29 before completing the walking tests. The MSIS-29 is a standardized self-evaluation form that requires patients to rank the impact of MS symptoms from 1 (no impact) to 5 (extreme) across various physical and psychological questions [28].

We selected a subset of MSIS-29 questions related to gait dysfunction and included only those patients with a score of 3 or higher (mild to moderate) for at least one question. 35 patients were excluded at this step. The average EDSS score for the remaining patients was 2.74 ± 2.06. Control participants were not required to complete the MSIS-29 questionnaire. The final dataset included 72 patients and gait data from 16 healthy controls. Table 2 shows the patients’ demographic and MSIS-29 information.

**Table 2:** Patient demographic and MSIS-29 information

Patient data features	Mean, variance
EDSS score	2.74 ± 2.06
Age	47.95 ± 9.8
Height (cm)	169.97 ± 8.06
Weight (kg)	82.2 ± 20.37
MSIS-29-Q4 Problems with your balance?	2.99 ± 0.94
MSIS-29-Q5 Difficulties moving about indoors?	2.14 ± 1.01
MSIS-29-Q6 Being clumsy?	2.76 ± 1.01
MSIS-29-Q7 Stiffness?	2.86 ± 1.15
MSIS-29-Q8 Heavy arms and/or legs?	2.90 ± 1.14
MSIS-29-Q9 Tremor of your arms or legs?	2.17 ± 1.17
MSIS-29-Q10 Spasms in your limbs?	2.29 ± 1.25
MSIS-29-Q11 Your body not doing what you want it to do?	2.39 ± 1.21

Patients and healthy controls walked at a comfortable pace across the instrumented walkway (Zeno Walkway, Protokinetics Haverton PA) measuring 90 × 420 cm, containing a matrix of embedded sensors with a spatial resolution of 1.27 cm and a resolution accuracy of ± 1.27 cm. Spatial measurements are provided as the (x,y) positions of activated sensors, which are converted to distances measured in cm. Time stamps recorded when each sensor was activated, measured in seconds.

### Data analysis and feature extraction

#### Deriving footprints from raw sensor data

The raw data from the walkway provides the time, X-coordinate, Y-coordinate, pressure level, foot type, foot count, footfall, and Pass Index for each sensor. We focused our analysis on two spectrums: time and location. If a sensor was detected multiple times at varying pressure intensity, only the time stamp for maximum pressure was selected. This temporospatial data collected allowed reconstruction of each pass across the walkway.

The raw spatial information was partitioned into left and right footfalls using a K-Means clustering [33] for each gait recording. The unsupervised clustering algorithm separated the *n* spatial coordinates into *k* individual footfalls, where each observation belongs to the cluster with the nearest centroid.

**Fig. 4:**
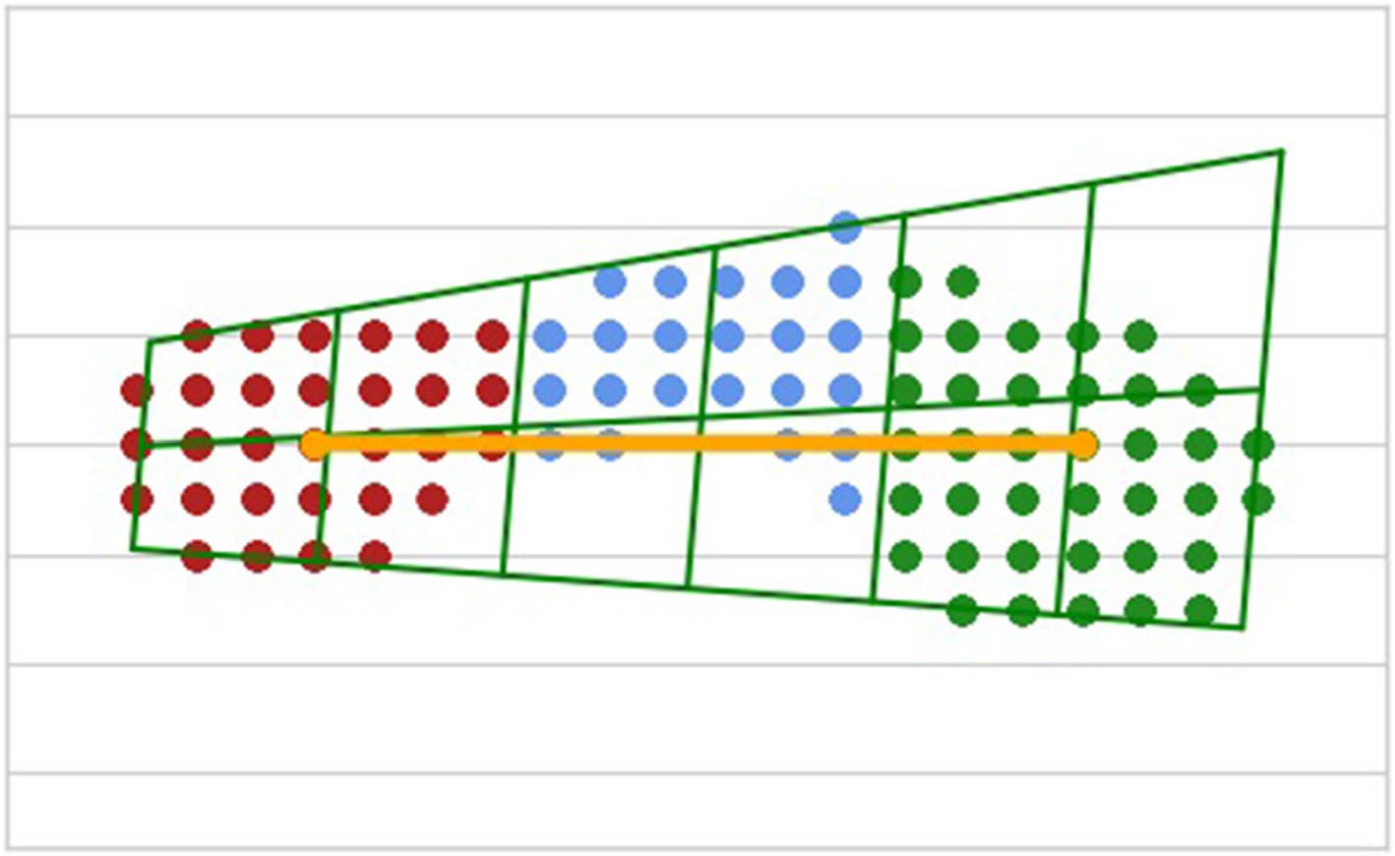
Footprint segmentation. Footprint showing heel (red), mid (blue), and fore (green) sections, as well as centerline of the foot (yellow) and the segmented quadrilateral enclosing the shape

For each footprint cluster, a quadrilateral was generated which enclosed the shape of the foot. This quadrilateral was then subdivided into three regions with individual sub-centroids, which provided further detail on the heel, mid, and fore sensors of the footprint. Figure 4 demonstrates how a footprint is segmented.

#### Standard gait features

After identifying the unique footfalls from the gait recording, an analysis was performed on each footfall, and standard gait parameters were extracted. These included step/stride length and width; toe in/out; step/stride time and velocity; single/double support time; and stance time.

Dimensions of foot length, width, and area are rarely documented as features in gait-related classification studies. Since these features were present in our data set, we included them to examine whether they could affect classification accuracy. The details regarding each parameter can be found in Table 3.

**Table 3:** Detail description for each standard gait parameter

Standard gait features		
Spatial features	Foot type	Descriptor for right or left foot
	Foot length (cm)	Measured as the distance between heel/fore centroids multiplied by 1.5
	Foot width (cm)	Measured as the distance across the midpoint of the subregion enclosing the fore section of the footprint
	Foot area (cm^2^)	Measured as the total activated area of the sensors involved in generating the footprint
	Toe angle	Measured as the angle between the line of progression (the line connecting the heel centers of two consecutive footprints of the same foot) and the midline of the footprint (the line connecting the heel and fore centroids of a given foot)
	Step length (cm)	Measured along the direction of the walkway, from the heel center of current footprint to heel center of previous footprint on opposite foot
	Step width (cm)	Measured from the midline midpoint of the current footprint to the midline midpoint of the previous footprint on the opposite foot
	Stride length (cm)	Measured on the line of progression between the heel points of two consecutive footprints of the same foot (left to left, right to right)
	Stride width (cm)	Measured as the vertical distance from midline midpoint of one footprint to the line formed by midline midpoints of two footprints of the opposite foot
	Base width (cm)	Measured as the vertical distance from heel center of one footprint to the line of progression formed by two footprints of the opposite foot
Temporal features	Step time (s)	The time elapsed from first contact of one foot to first contact of the opposite foot
	Stride time (s)	The time elapsed between the first contacts of two consecutive footfalls of the same foot. It is measured in seconds
	Stride velocity (cm/s)	Obtained by dividing the stride length by the stride time
	Step velocity (cm/s)	Obtained by dividing the step length by the step time
	Single support time (s)	The time between the last contact of the current footfall to the first contact of the next footfall of the same foot
	Double support time (s)	Measured as the time between the heel contact of next footfall to toe-off of the current (and opposite) footfall
	Stance time (s)	Measured as the time between first contact and last contact of the same foot

#### New feature design

**Fig. 5:**
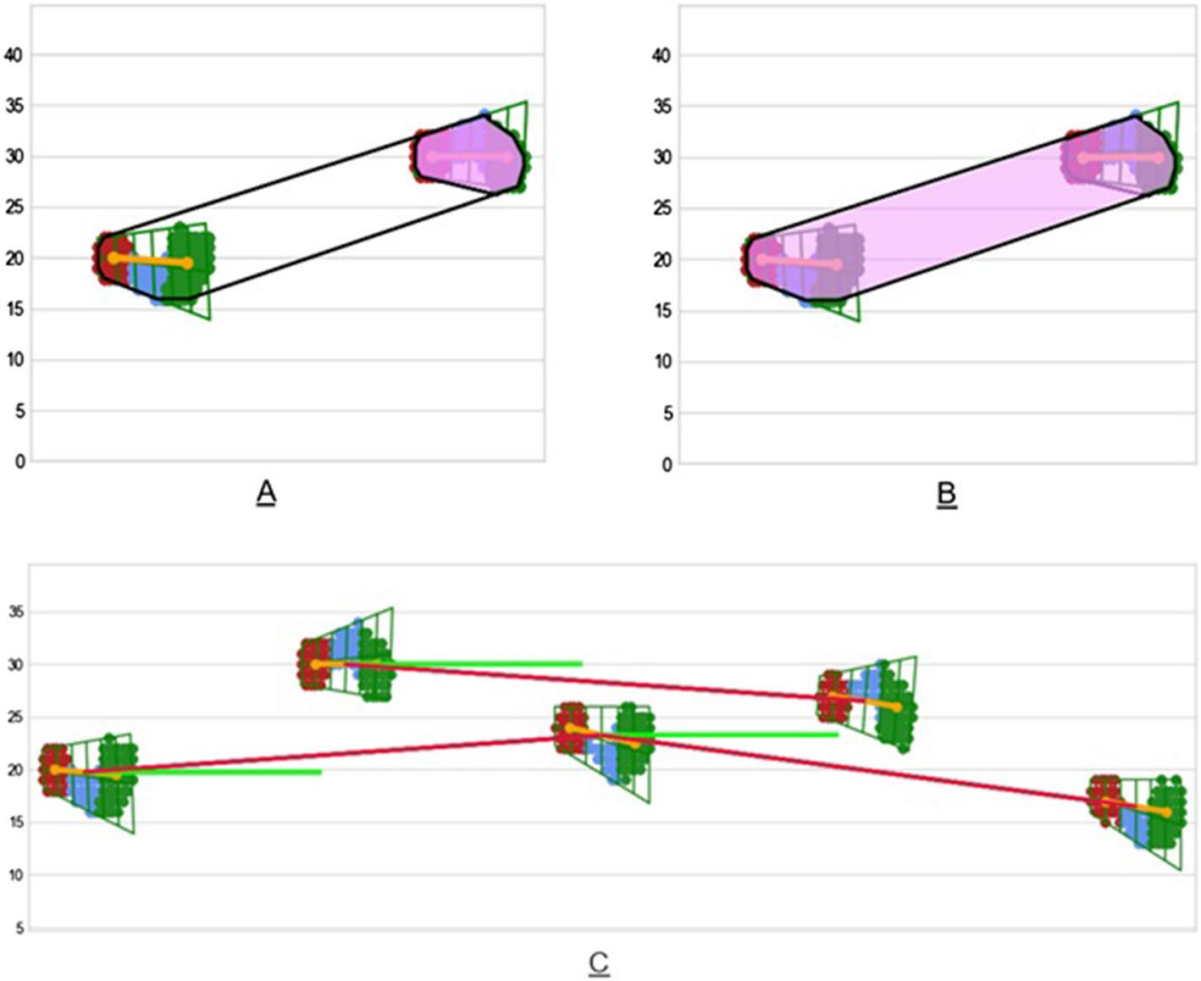
**A** The light pink shaded region shows the hull area for a single footfall. **B** The light pink shaded region shows the BOS area between two successive footfalls. **C** The light green line represents the normal (desired) line of progression, the red line represents the actual line of progression between two consecutive footfalls of the same foot. The angle between the desired and actual lines is the line of progression deviation angle

New parameters were designed and calculated from the walkway data (Fig. 5 and Table 4). As far as we are aware these features have not yet been rigorously tested in a patient/controls classification setting.

**Table 4:** Detailed descriptions of newly designed features

New feature design	
Toe direction	Standard toe angle is recorded by the walkway as a signed value. We split the original toe angle value into two features: magnitude and direction. We keep the absolute value of deviation in toe angle and store the toe direction as a binary categorical feature, with 0 denoting negative toe angle and 1 denoting toe angles greater than or equal to zero
Hull area	To better approximate the actual shape of the footprint, we calculated the convex hull enclosing the point cloud for each footprint. The hull area is the enclosed area of the line segments bounding the footprint tightly in a convex hull. Figure 5A shows the hull area
BOS area	In gait, the BOS [23] is commonly measured as a one-dimensional length. We construct a convex polygon enclosing two footprints (a footprint and its preceding print) and then use this to approximate the area of the BOS. Figure 5B shows the picture of BOS area
LOP deviation angle	The deviation angle from an expected normal line of progression. A LOP is the line connecting the heel centers of two consecutive footfalls of the same foot. Ideally, the patient should be walking parallel with the walkway. We measure the deviation angle as the angle between: - a line that starts on a foot and runs parallel to the walkway (the 'normal LOP') - a line that starts at the center of the same foot, and stops at the center point of the next footfall of the same foot (the actual LOP) Figure 5C shows the picture of line of progression deviation angle

#### Feature sets design for classification

Two feature sets, namely the standard feature set and the augmented feature set, were designed for the classification task. The standard set included the step time, stride time, step velocity, stride velocity, single support time, double support time, stance time, foot type, toe angle signed, step length, step width, stride length, stride width, and base width.

The augmented set included all the features from the standard set, as well as additional parameters of foot length, foot width, foot area, hull area, LOP deviation angle, BOS area, toe angle, and toe direction.

### Machine learning process

#### Data balancing

With a patient-to-control ratio of approximately 6:1, we performed balancing on the target classes before proceeding with classification analysis [34]. The training data set were balanced using a synthetic minority oversampling technique (SMOTE). SMOTE synthesizes a new sample by randomly choosing a data point from a line segment in the feature space, formed by a minority class sample m and one of m’s k-nearest neighbors (usually k = 5, both randomly chosen); then this process is repeated till the two classes’ data are balanced [35].

#### Data normalization

The numerical data collected exhibited a variety of ranges between different features and participants and thus required scaling. The resulting numerical data columns were proportionally scaled to exhibit zero mean and unit variance. The mean and variance calculated from the training set were applied to both the training and testing datasets.

In addition to proportionally scaling the ranges for each feature, it was also necessary to normalize the measurements for foot length, foot width, foot area, and hull area. This was accomplished by dividing the individual parameter measurement for each patient by the patient’s height (cm).

#### Feature selection

**Fig. 6:**
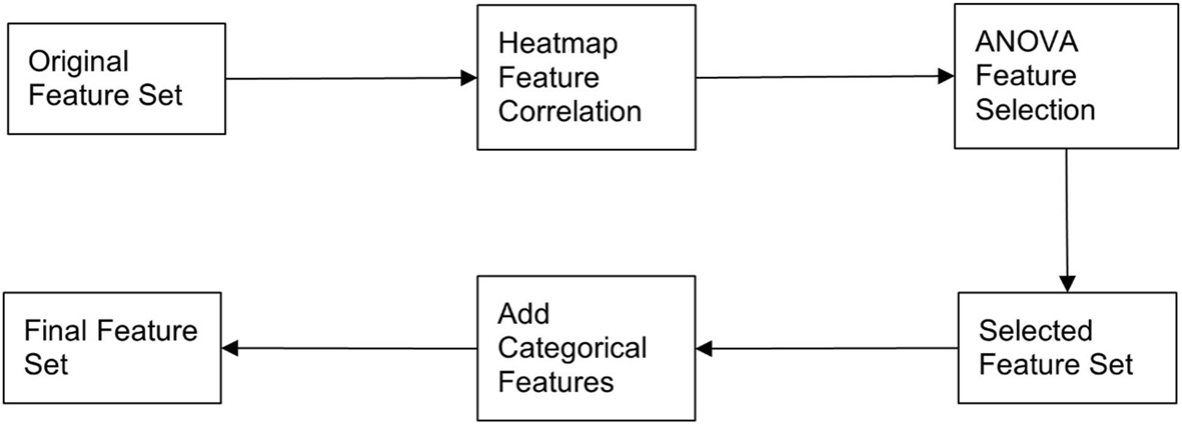
Feature selection process

**Fig. 7:**
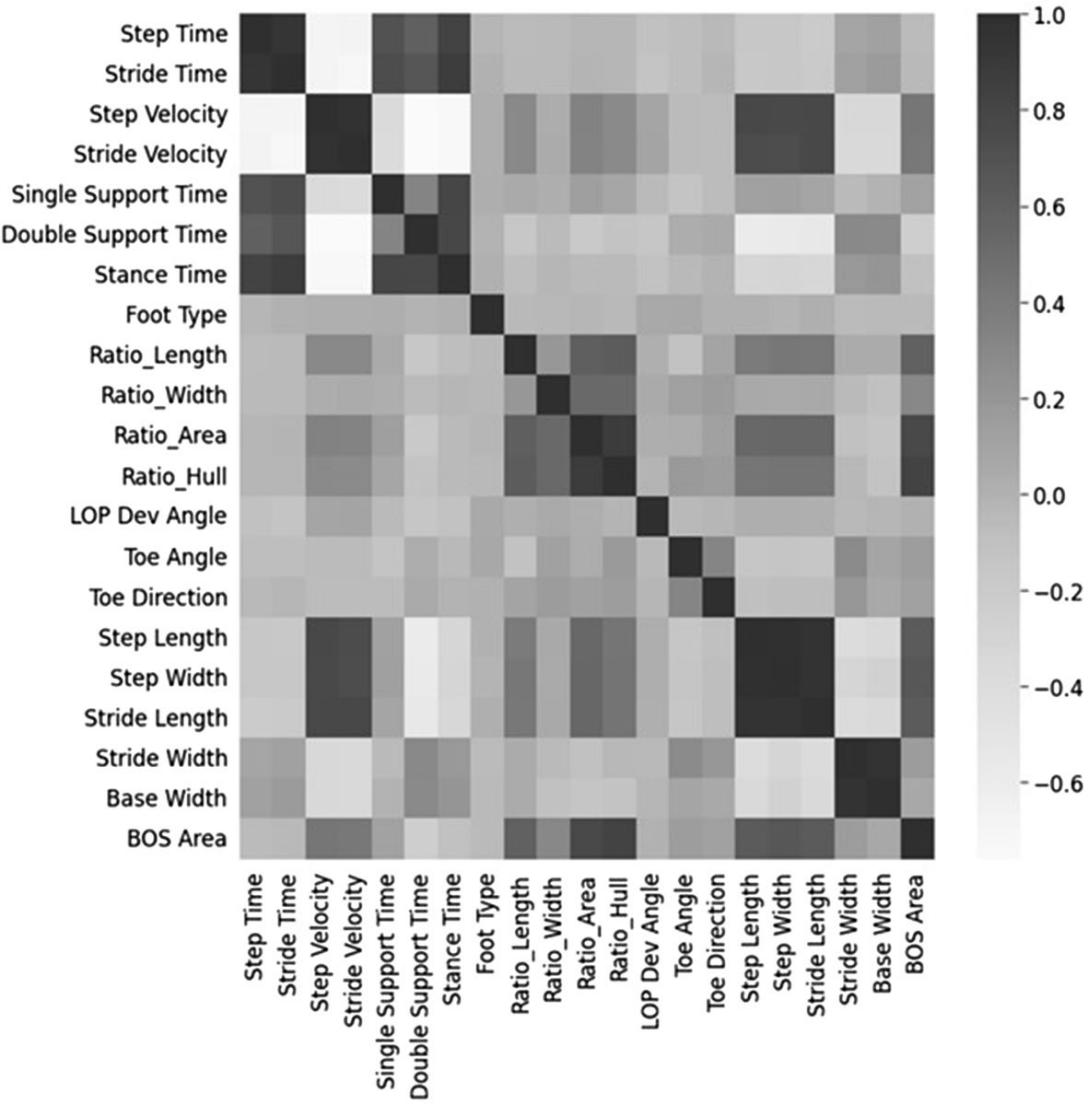
Heatmaps for correlations between features in the standard and augmented feature sets. Heatmap regions that are increasingly dark show areas of higher correlation, vice versa

Figure 6 shows the process of feature selection. Reducing correlation among the numerical features is important for reducing prediction bias, speeding up the training process for the models, limiting unnecessary noise in the data, thus improving the overall effectiveness of the classifier. Pearson correlation was used to reduce the number of dependent features and a heatmap was used to visualize the correlations between features of training set. The resulting feature correlation matrix contained scores ranging from -1, strong negative correlation, to + 1, strong positive correlation, with a score of 0 denoting no correlation between the features. Our study used a removal threshold of − 0.8/0.8 for feature correlation. The heatmap determined the interdependence of all numerical features shown in Fig. 7.

The heatmap shows a strong positive correlation between the ‘step’ and ‘stride’ parameter sets (*r* > 0.8), as well as the base width and stride width. Stride time, stride velocity, and base width were excluded from further analysis to reduce the interdependence among the features.

Once the highly correlated features were removed, feature selection was performed on both the standard feature set and the augmented feature set, respectively, to determine which features provided the strongest response on the target variable, and to determine the optimal size of each set. The goal was to build two optimized sets of features (standard and augmented) which were used in the training and testing process.

Analysis of variance F-test statistics (ANOVA) was used on the training set to choose a subset of numerical features that had the most impact on the response variable. ANOVA gives each feature a score, with higher scores representing stronger features that have greater unexplained variance in prediction. When the features were ranked by their F-statistic score, it was then necessary to choose the size of the final set.

To determine the optimal size of this final feature set, all features were ranked by ANOVA score. Then, for each possible size s_i_ of the final set [1, 2, …n features], a fivefold cross-validation strategy with a SVM classifier was used to get the prediction accuracy for each size s_i_. The average prediction accuracy was collected for each size s_i_, and the optimal size was chosen with the highest score.

Since the categorical features are not included in the correlation or feature selection process, it is necessary to reintroduce these to the final feature set when the numerical processes are completed.

The standard set was initialized with 11 input features, from which the ANOVA algorithm suggested an optimal subset of 9 features. Step time was dropped as it had the lowest ANOVA F-statistic of the original group. When numerical feature selection was completed, the categorical feature foot type was reintroduced, resulting in the final standard set.

The augmented feature set was created from the same base features as the standard set, and these were complemented with hull area, BOS area, LOP deviation angle, toe angle magnitude, foot length, foot width, and foot area. Once completed, the ANOVA algorithm suggested an optimal size of 13 best features in the augmented set. The same features as the training set were dropped in the testing set. Tables 5 and 6 provide detail F-statistic score for optimal features.

**Table 5:** F-statistic score for optimal standard set features

Features	Feature score
Stride length	974.54
Step length	924.42
Step width	862.72
Stride width	330.42
Step velocity	243.75
Single support time	37.58
Double support time	25.77
Toe angle signed	4.89
Stance time	4.10

**Table 6:** F-statistic score for optimal augmented set features

Features	Feature score
Stride length	977.28
Step length	934.28
Step width	874.32
Foot area	374.92
BOS area	281.11
Stride width	265.59
Step velocity	250.78
Hull area	222.84
Foot length	91.88
Single support time	67.70
Double support time	29.05
Foot width	19.32
Toe angle unsigned	2.37

#### Machine learning algorithms

We tested the separability of the target classes using three general classification algorithms. LR [36], SVM [37], and XGB [38] were selected as they represent three well known methods of classification; probability, hyperplane polarity, and boosted decision-tree ensembles. Given a set of input features, each model was studied for its ability to categorize footprints as belonging to an MS patient or a healthy control through a range of classification scoring metrics.

LR is arguably the most popular binary classifier in machine learning. It relies on a logistic function into which input values x are combined linearly using weights or coefficient values to predict an output value y which is modeled as a binary categorical response [36].

SVM attempts to define a hyperplane boundary in an N-dimensional space, where N equals the number of input features. While many hyperplanes may exist in this space, SVM attempts to find the optimal plane that maximizes the separation of both classes. Additional points can then be classified as belonging to class 0 or 1 depending on the side of the optimal hyperplane that they occupy [37].

XGB is an optimized distributed gradient boosting library introduced by Chen & Guestrin in 2016 [38]. Applied to an ensemble of decision trees, boosting describes the combination of many weak learners into one accurate prediction algorithm. XGB utilizes the concept of gradient tree boosting while introducing regularization parameters to reduce overfitting.

#### Training and evaluation

To further reduce overfitting, we employed a grouped fivefold cross-validation strategy when training each model. All rows in the dataset were grouped according to the date of the patient visit and given a unique identifier. These groups remained intact throughout train/test validation splitting, and no group was permitted to appear in two different folds. In this fashion, the same participant’s data were not used simultaneously in training and testing sets.

Each model in the study has a unique set of hyperparameters that must be tuned to provide the best result. We used a standard grid search method on training data to test each model across a range of hyperparameter settings and selected the best parameter values for each. A summary of the tested parameter values for each model, along with the optimal hyperparameter settings for this data set, can be found in Tables 7 and 8.

**Table 7:** Hyperparameter options for each model

Algorithms	Hyperparameter options
LR	'solver': ['newton-cg','lbfgs', 'liblinear'], 'penalty': ['l1', 'l2', 'elasticnet'], 'C': [1000, 100, 10, 1.0, 0.1, 0.01]
SVM	'kernel': ['poly', 'rbf', 'sigmoid'], 'C': [5, 3, 1.0, 0.5, 0.1], 'degree': [3–5]
XGB	'max_depth': [2, 3], 'eta': [0.3, 0.4], 'objective': ['binary:logistic', 'binary:logitraw', 'binary:hinge']

**Table 8:** Hyperparameters used by each algorithm to train the model

Algorithms	Optimal hyperparameters (standard set)	Optimal hyperparameters (augmented set)
LR	'C': 1.0, 'penalty': 'l2', 'solver': 'newton-cg'	'C': 1.0, 'penalty': 'l1', 'solver': 'liblinear'
SVM	'C': 3, 'degree': 3, 'kernel': 'rbf'	'C': 5, 'degree': 3, 'kernel': 'rbf'
XGB	'eta': 0.3, 'max_depth': 3, 'objective': 'binary:logistic'	'eta': 0.3, 'max_depth': 3, 'objective': 'binary:logitraw'

The number of true positive (TP), true negative (TN), false positive (FP), and false negative (FN) [39] predictions were calculated for each model, and a range of standard classification metrics were calculated to gauge the model effectiveness. Score metrics are explained in Table 9.

**Table 9:** Accuracy, precision, recall and F1 score explanation. TP, TN, FP, and FN are true positive, true negative, false positive, and false negative, respectively

Accuracy (%)	The total number of correct predictions out of the total number of all predictions	$\frac{\text{TN}+\text{TP}}{\text{TN}+\text{TP}+\text{FN}+\text{FP}}*100\text{\%}$
Precision (%)	Or positive predictive value. represents the proportion of true positive predictions to all actual positives	$\frac{\text{TP}}{\text{TP}+\text{ FP}}*100\text{\%}$
Recall (%)	Or sensitivity. measures the proportion of true positives to the total number of actual positives	$\frac{\text{TP}}{\text{TP}+\text{FN}}*100\text{\%}$
F1 Score (%)	The weighted harmonic mean of precision and recall	$2* \frac{\text{Precision}*\text{sensitivity}}{\text{Precision}+\text{sensitivity}}*100\text{\%}$

ROC and PR curves were also generated for each model. The area under these curves can be assessed as another measure for determining the predictive capability of the model.

## Data Availability

The datasets used and or analyzed during the current study are available from the corresponding authors on reasonable request.

## Abbreviations

ANOVA: Analysis of variance
AUPRC: Area underneath precision-recall curve
AUROC: Area underneath receiver operating characteristic curve
BOS: Base of support
EDSS: Expanded disease severity scale
FN: False negative
FP: False positive
HITMS: Health innovation team in multiple sclerosis
LOP: Line of progression
LR: Logistic regression
MS: Multiple sclerosis
MSIS-29: Multiple sclerosis impact scale
PR: Precision recall
ROC: Receiver operating characteristic curve
SVM: Support vector machine
TN: True negative
TP: True positive
XGB: Extreme gradient boosting
